# Quackery Extraordinary

**Published:** 1854-01

**Authors:** 


Quackery Extraordinary.—We were somewhat, but not much, surprised upon seeing a column of the Quack Advertising Department of the N. Y. Tribune devoted to a grandiloquent exposition of the marvelous merits of a new series of nostrums, got up and heralded forth by a certain Dr. James McClintock, late Professor of three departments, at once, in a sort of fourth rate medical college at Philadelphia—formerly professor of a number of different things in two or three other medical colleges—member of quite a number of medical societies—and who winds up his list of titles with three individual &c.'s. How was he a *late* member of the American Medical Association? Was he expelled, or did he resign, in anticipation of his recent treachery to the honor of the medical profession?

We say we were not much surprised at the above development, for our previous knowledge of his character and career led us to expect that he would, sooner or later, come to this general pool of dilapidated doctors, and comminuted migratory professors. We have occasion to know that his previous positions of some apparent importance, were obtained by accidental circumstances, and that he failed to sustain himself from a lack of professional integrity. Of course this late bold secession to the ranks of quackery is no sudden movement,—it does no violence to his nature—the disease has only assumed a different and more natural phase, as the eruption is now fully out. The fulsome and bloated style of his elongated show-bill is so thoroughly impregnated with the spirit of quackery, that the theme must have long been a favorite subject of contemplation. Even the small cunning of his shallow disguises, only shows the desire to make a deeper and more effectual thrust. The allusions to "specifics, elixirs, liver complaints, vegetable remedies," &c., are quite in the old way of his illustrious prototypes, Brandreth & Co., although *they* never magnified the "cheapness" of their wares, and then stated that they were made "regardless of expense." He states, by way of apology, that he is driven to this course, to save time from answering the numerous applications for his medicines, from the crowding millions of his former pupils and patients. But could not the profound ex-Professor have favored the ignorant medical profession with some knowledge of his tympanitic discoveries? or, would no one of the forty medical journals in the United States answer his purpose?

But the *manner* of his defection demands but little notice. The prominent fact, naked and alone, is enough to call down the heartiest contempt of every enlightened medical mind. The individual who sells *himself* to a New York druggist, in the same bargain with a dozen ordinary prescriptions, only declares by that act, that he has at length reached the level towards which his nature had long been carrying him. There are perhaps but one or two other steps necessary to complete this rapid *descensus averni.* A few months of public ostentation, in the line of spiritual rappings, and then the final performance of opening a popular water cure establishment.

Seriously, it is a saving principle, that the men who thus finally turn traitors to the position they have occupied for years, are the very men whose nature and character have always prevented them from achieving any eminence in their earlier pursuits. Much as we may regret any accession to the forces of quackery, still we are quite willing to see a thorough ventilation of the medical profession; for nothing more promotes the healthy action and vitality of the medical community proper, than an occasional expurgation. There are some others, that we wot of, who are following the above example, and who will, in good time, confer a favor by relieving the medical world of morbid accumulations.

We design in the above, no particular attack upon the individual in question. It is *he* who has publicly made a most hostile attack upon the honor and character of the medical profession, throughout the United States, and we are but in the path of duty, in doing what we may to expose and denounce such recreancy.

